# Rezidiv einer akuten lymphoblastischen B‐Zell‐Leukämie oder CAR‐T‐Zell‐bedingte Hautveränderungen – eine relevante diagnostische Herausforderung

**DOI:** 10.1111/ddg.15931_g

**Published:** 2026-02-05

**Authors:** Farzan Solimani, Amrei Dilling, Konrad Heisterkamp, Frederik Damm, Olaf Penack, Christian Oberender, Philipp Le‐Coutre, Martina Rudelius, Kamran Ghoreschi, Alexander Nast

**Affiliations:** ^1^ Klinik für Dermatologie Venerologie und Allergologie Charité – Universitätsmedizin Berlin Körperschaft der Freien Universität Berlin der Humboldt‐Universität zu Berlin und des Berlin Institute of Health, Berlin; ^2^ Klinik für Hämatologie Onkologie und Tumorimmunologie Charité – Universitätsmedizin Berlin Körperschaft der Freien Universität Berlin und der Humboldt‐Universität zu Berlin Campus Virchow Klinikum, Berlin; ^3^ Klinik für Hämatologie Onkologie und Tumorimmunologie Charité – Universitätsmedizin Berlin Körperschaft der Freien Universität Berlin und der Humboldt‐Universität zu Berlin Campus Charité Mitte, Berlin; ^4^ Institut für Pathologie Ludwig‐Maximilians‐Universität München

Sehr geehrte Herausgeber,

Die *Chimeric Antigen Receptor* (CAR)‐Therapie bringt revolutionäre therapeutische Konzepte in die klinische Praxis und ermöglicht es Ärzten, bestimmte Zellen gezielt zu eliminieren.^1^ CAR‐T‐Zellen basieren auf gentechnisch veränderten T‐Zellen, die selektiv gegen bestimmte Antigene gerichtet sind.^1^ Die steigende Zahl von Patienten, die CAR‐T‐Therapien erhalten, führt auch dazu, dass Kliniker immer häufiger auf seltene unerwünschte Ereignisse aufmerksam werden. Die kutanen Nebenwirkungen der CAR‐T‐Therapie sind kaum dokumentiert und werden nur selten beschrieben, so dass es für Kliniker schwierig ist, zwischen dem Wiederauftreten der Krankheit und sekundären kutanen Toxizitäten zu unterscheiden. Ein aktueller Fall aus unserer Abteilung veranschaulicht die klinischen und diagnostischen Herausforderungen von CAR‐T‐Zell‐induzierten kutanen unerwünschten Nebenwirkungen.

Bei einem 50‐jährigen Mann wurde eine *BCR‐ABL*‐ und *KMT2A*‐negative akute lymphoblastische B‐Zell‐Leukämie (B‐ALL) diagnostiziert und zunächst mit einem chemotherapeutischen Induktionsschema (Dexamethason, Cyclophosphamid, Daunorubicin, Peg‐Asparaginase, Cytarabin) und Rituximab behandelt. Dies löste einen Antithrombin‐III‐Mangel aus, und die Behandlung wurde auf Wunsch des Patienten abgebrochen. Acht Monate später stellte sich der Patient wegen des Auftretens infiltrierter Plaques am Hinterkopf in unserer Hautambulanz vor. Die histologische Untersuchung zeigte ein noduläres, teilweise diffuses blastisches Infiltrat mit einem Profil aus CD19^+^, PAX5^+^ und CD3^–^ sowie einem hohen Ki67‐Proliferationsindex (80%) (ABBILDUNG 1a). Es wurde ein Rezidiv der B‐ALL diagnostiziert. Achtzehn Monate später wurde eine Salvage‐Therapie mit Blinatumomab, einem bispezifischen monoklonalen Antikörper (mAb), der CD3 und CD19 erkennt und verbindet, begonnen, jedoch kurz darauf aufgrund einer generalisierten Serositis abgebrochen. Nach gründlicher Aufklärung über die verfügbaren Optionen lehnte der Patient eine allogene Transplantation ab und entschied sich für eine Anti‐CD19‐CAR‐T‐Behandlung, die 4 Monate später begonnen wurde. Nach Überbrückungstherapie mit Inotuzumab Ozogamicin (einem humanisierten mAb gegen den B‐Zell‐Marker CD22 in Verbindung mit einem zytotoxischen Wirkstoff) und Konditionierung mit Fludarabin und Cyclophosphamid erhielt der Patient 1 × 10⁶ CAR‐T‐Zellen/kg (Brexucabtagene Autoleucel, ein autologes CD19‐gerichtetes CAR). Ein Zytokinfreisetzungssyndrom (*cytokine release syndrome*; CRS) Grad II wurde umgehend mit Tocilizumab (einem anti‐IL‐6‐Rezeptor‐mAb) behandelt. Acht Monate nach der CAR‐T‐Infusion stellte sich der Patient erneut in unserer Abteilung vor, weil schmerzlose, nicht juckende Makulae und leicht infiltrierte Plaques und Knötchen am Hinterkopf, am Bauch und am Knie auftraten (Abbildung 2). Es gab keine Hinweise auf eine weitere organbezogene Toxizität. Die Anamnese schloss auch eine Kontaktallergie, ein virales Exanthem oder eine impfstoffbedingte Reaktion aus. Das Differenzialblutbild zeigte eine leichte Abnahme der zirkulierenden Neutrophilen und eine Zunahme der Monozyten, während sich bei den zirkulierenden CD4/CD8‐T‐ und *Natural‐Killer* (NK)‐Zellen‐Populationen keine pathologischen Veränderungen zeigten. Zusätzlich berichtete der Patient über Morgensteifigkeit, Gelenkschmerzen und Myalgien. Wir vermuteten ein Rezidiv der B‐ALL und untersuchten Biopsate aus der Bauch‐ und Hinterhauptsregion histologisch. Die Histologie ergab eine dichte lymphozytäre und eosinophile Infiltration mit perivaskulärer Akzentuierung. Die lymphozytäre Infiltration bestand fast ausschließlich aus gemischten CD4/CD8‐CD30^–^‐T‐Zellen (Abbildung 1b–f), während B‐Zelllinien‐spezifische Marker (CD20, CD19, Pax5) nur wenige positive Zellen aufwiesen, was ein B‐Zell‐Malignom ausschloss (Abbildung 1g–h). Auch Ki67 war nicht signifikant erhöht und die terminale Desoxynukleotidyltransferase (TdT, ein Marker für lymphatische Lymphome und Leukämien) war negativ (Abbildung 2). Die Untersuchung der Klonalität der schweren Immunglobulinkette (IgH, Regionen FR1‐4) und des T‐Zell‐Rezeptors (TCR)γ (Vγ/J, Vγ/Jp) zeigte ein polyklonales IgH‐Repertoire und keine TCR‐Klonalität. Diese Ergebnisse sprachen gegen eine B‐ALL oder sonstige hämatoonkologische Erkrankungen, und wir stellten die Diagnose einer CAR‐T‐Zell‐induzierten pseudo‐lymphatischen Hautreaktion. Die Behandlung mit hochwirksamen topischen Steroiden hat bisher nur leichte Besserung gebracht. Die Läsionen besserten sich in den ersten 3 Monaten deutlich, danach zeigten sie eine behandlungsresistente Restentzündung. Zwölf Monate nach der CAR‐T‐Zell‐Behandlung ist der Patient bei den Nachuntersuchungen in Bezug auf B‐ALL weiterhin in Remission und weist weiterhin leichte symptomfreie erythematöse Restläsionen auf.

**ABBILDUNG 1:**
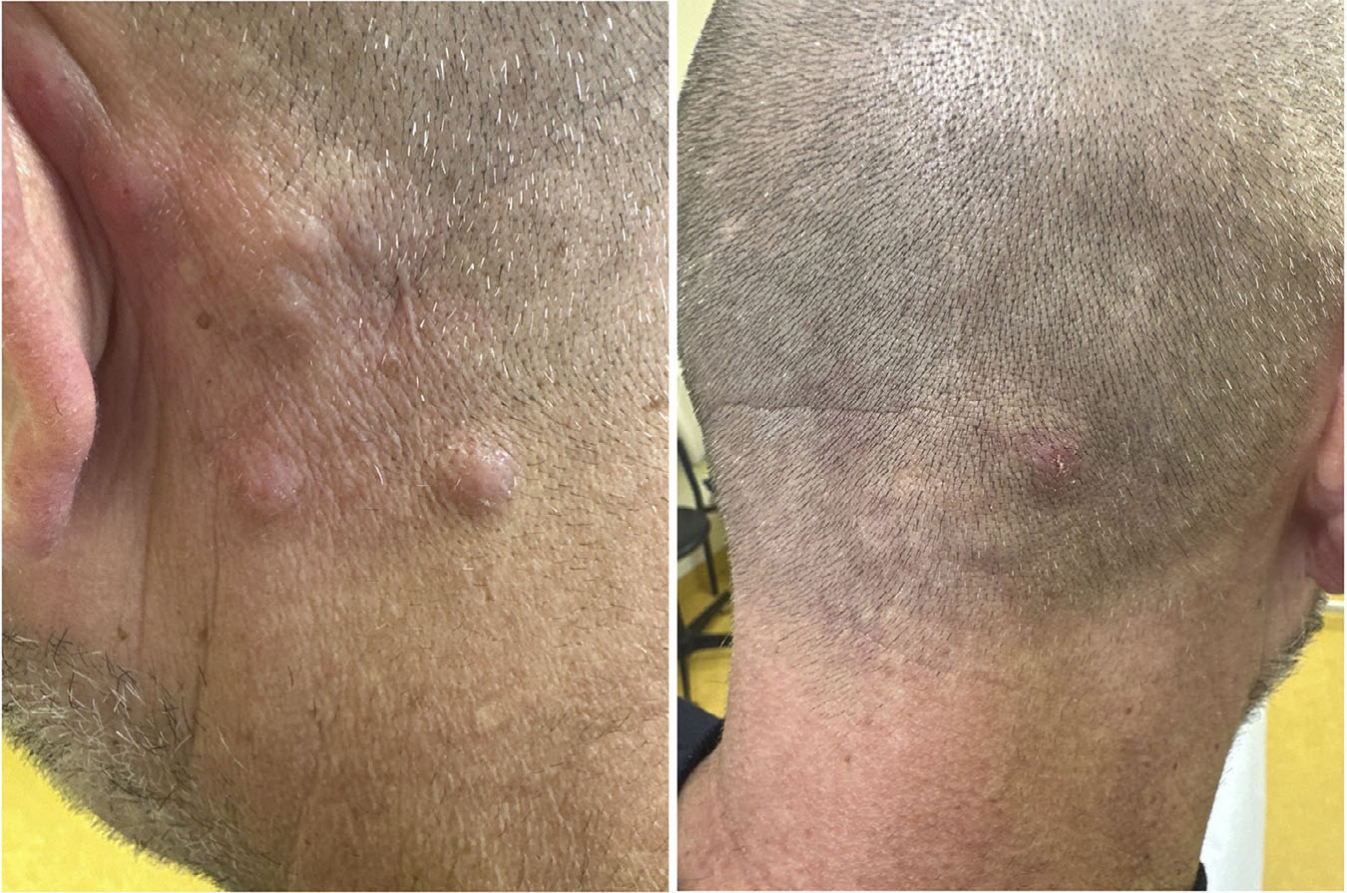
(a) Klinische Manifestationen der durch CAR‐T‐Zellen induzierten Hautveränderungen bei einem 50‐jährigen männlichen Patienten, der wegen akuter lymphoblastischer B‐Zell‐Leukämie (B‐ALL) mit Anti‐CD19‐CAR‐T‐Zellen behandelt wurde.

**ABBILDUNG 2:**
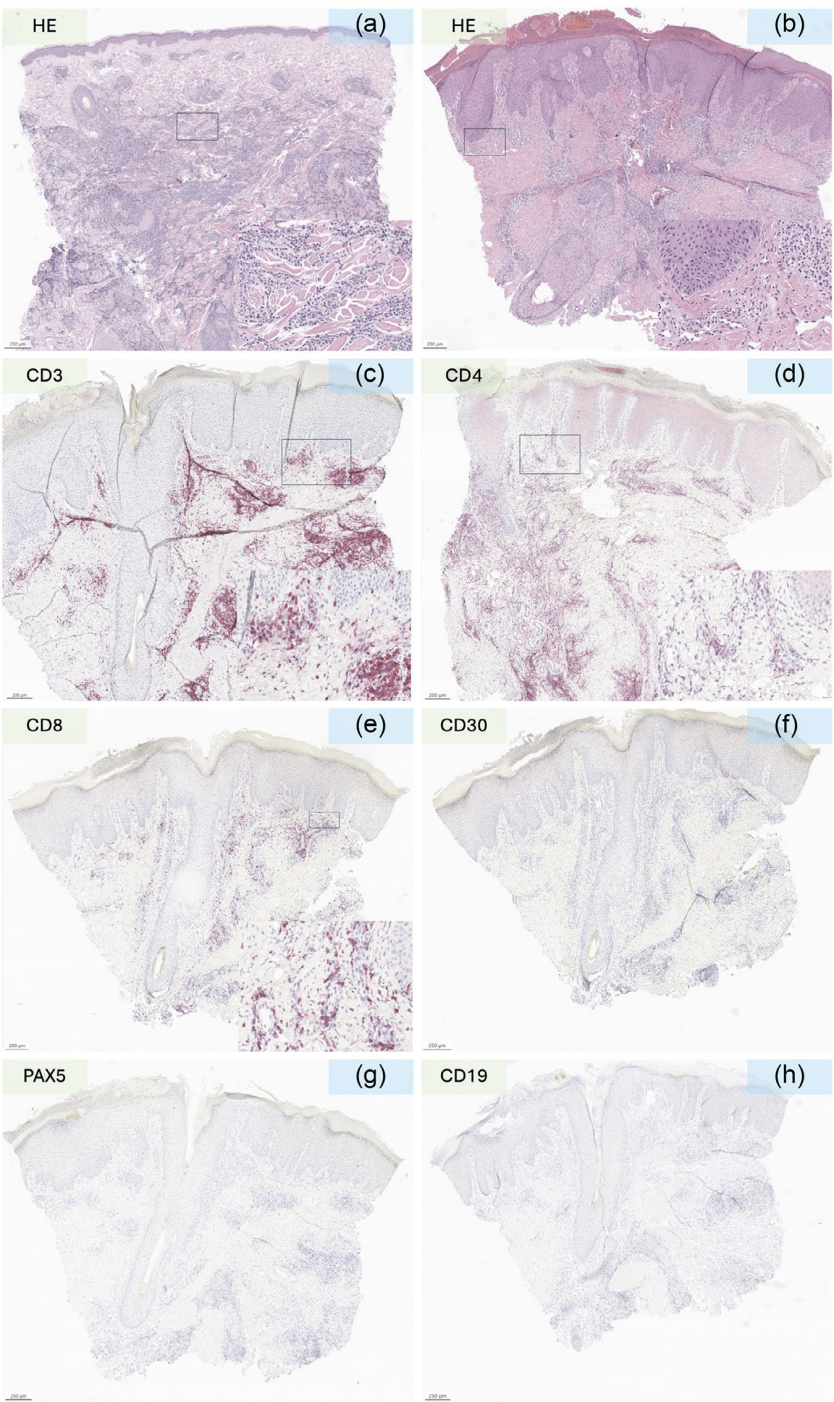
(a) Hämatoxylin‐ und Eosin‐Färbung (HE) der B‐ALL‐induzierten Leukaemia cutis vor der Behandlung mit Anti‐CD19‐CAR‐T‐Zellen; (b) HE‐Färbung der Anti‐CD19‐CAR‐T‐induzierten kutanen Nebenwirkung; (c–f) T‐Zell‐spezifische Färbung (CD3, CD4, CD8, CD30) zeigt ein gemischtes CD30‐negatives CD4/CD8‐Lymphozyten‐Infiltrat; (g, h) PAX5‐ und CD19‐Färbung mit nur wenigen B‐Zellen in der betroffenen Haut. Maßstabsbalken = 250 µm (a, f–h) oder 200 µm (b–e), wie in der unteren linken Ecke jeder Färbung angegeben. (a–e) Details des dermalen zellulären Infiltrats in den unteren rechten Kästen; Maßstabsbalken = 20 µm.

Während das Zytokinfreisetzungssyndrom und das *immune effector cell‐associated neurotoxicity syndrome* (ICANS) als Nebenwirkungen gut beschrieben sind, müssen kutane Manifestationen noch genauer untersucht werden und sind klinisch herausfordernd, insbesondere da sie eine Leucaemia cutis imitieren können. Eine kürzlich durchgeführte Studie über klinische CAR‐T‐Studien zeigte, dass bei bis zu 35% der teilnehmenden Patienten leichte bis mittelschwere Hautreaktionen auftreten.^2^ Fehlende dermatologische Erfahrung könnte höhere Raten verschleiern. Für Ärzte ist es entscheidend, Rezidive zuverlässig auszuschließen. Wir führten eine immunhistochemische Untersuchung und eine Klonalitätsbeurteilung durch, um eine Leucaemia cutis auszuschließen. Andere Gruppen verfolgten andere Ansätze. Eine Gruppe untersuchte mit Hilfe der Durchflusszytometrie Blasenflüssigkeit und T‐Zellen aus Hautläsionen eines Patienten mit bullöser Eruption; diese zeigte CD3‐gemischte CAR‐T‐positive und CAR‐T‐negative Zellen.^4^ In einer Studie zur CD30‐CAR‐T‐Zelltherapie bei Hodgkin‐Lymphom wurden die Hautreaktionen histologisch und mittels quantitativer Polymerasekettenreaktion analysiert, wobei die Expression des CD30‐CAR‐Transgens nachgewiesen wurde.^5^ Der zugrunde liegende Immunpathomechanismus für CAR‐T‐induzierte Hautreaktionen muss noch geklärt werden. Bei unserem Patienten trat die Hautreaktion interessanterweise in der Okzipitalregion auf, wo der Patient ursprünglich eine Leucaemia cutis entwickelte. Dies könnte auf eine mögliche initiale Reaktion auf maligne residente B‐Zellen als *primum movens* für die Hautinfiltration hinweisen. In Einklang mit diesem Konzept beschrieben Hagen et al. kürzlich eine neue Form der CAR‐T‐bedingten Organtoxizität (*local immune effector cell‐associated toxicity syndrome*; LICATS) bei Patienten mit Autoimmunerkrankungen, die aufgrund der CAR‐T‐vermittelten B‐Zell‐Abtötung Entzündungsreaktionen in den betroffenen Organen entwickelten. Die gemeldeten kutanen Läsionen ähneln denen, die bei unserem Patienten beobachtet wurden und die ebenfalls durch die Abtötung von B‐Zellen in der Haut verursacht wurden.^6^

## DANKSAGUNG

Open access Veröffentlichung ermöglicht und organisiert durch Projekt DEAL.

## INTERESSENKONFLIKT

Keiner.
